# Multi-Step Enzymatic Synthesis of 1,9-Nonanedioic Acid from a Renewable Fatty Acid and Its Application for the Enzymatic Production of Biopolyesters

**DOI:** 10.3390/polym11101690

**Published:** 2019-10-15

**Authors:** Hyun-Ju Lee, Young-Seo Kang, Chae-Yun Kim, Eun-Ji Seo, Sang-Hyun Pyo, Jin-Byung Park

**Affiliations:** 1 Department of Food Science & Engineering, Ewha Womans University, Seoul 03760, Korea; leehyunju430@gmail.com (H.-J.L.); kys93524@gmail.com (Y.-S.K.); cy1125h@gmail.com (C.-Y.K.); sej3586@ewhain.net (E.-J.S.); 2 Biotechnology, Department of Chemistry, Center for Chemistry and Chemical Engineering, Lund University, SE-22100 Lund, Sweden; sang-hyun.pyo@biotek.lu.se

**Keywords:** whole-cell biocatalyst, alcohol/aldehyde dehydrogenase, α,ω-dicarboxylic acids, *Corynebacterium glutamicum*, polyesterification, polyester

## Abstract

1,9-Nonanedioic acid is one of the valuable building blocks for producing polyesters and polyamides. Thereby, whole-cell biosynthesis of 1,9-nonanedioic acid from oleic acid has been investigated. A recombinant *Corynebacterium glutamicum*, expressing the alcohol/aldehyde dehydrogenases (ChnDE) of *Acinetobacter* sp. NCIMB 9871, was constructed and used for the production of 1,9-nonanedioic acid from 9-hydroxynonanoic acid, which had been produced from oleic acid. When 9-hydroxynonanoic acid was added to a concentration of 20 mM in the reaction medium, 1,9-nonanedioic acid was produced to 16 mM within 8 h by the recombinant *C. glutamicum*. The dicarboxylic acid was isolated via crystallization and then used for the production of biopolyester by a lipase. For instance, the polyesterification of 1,9-nonanedioic acid and 1,8-octanediol in diphenyl ether by the immobilized lipase B from *Candida antarctica* led to formation of the polymer product with the number-average molecular weight (*M*_n_) of approximately 21,000. Thereby, this study will contribute to biological synthesis of long chain dicarboxylic acids and their application for the enzymatic production of long chain biopolyesters.

## 1. Introduction

Oils and fatty acids are one of the representative renewable biomass [1,2]. For instance, 189 million metric tons of vegetable oils were produced worldwide in 2013–2014 according to a USDA report (2015) [3]. In addition, production of microalgae oils from carbon dioxide have been extensively investigated recently [4,5,6].

Oils and fatty acids can be used as starting materials for the production of a variety of monomers for the polymers such as polyesters and polyamides [7,8,9,10,11,12]. For example, microbial production of ω-hydroxycarboxylic acids, α,ω-dicarboxylic acids, and ω-aminocarboxylic acids from fatty acids and fatty acid methyl esters have been reported [12,13,14,15]. 1,12-Dodecanedioic acid was produced from lauric acid methyl ester (dodecanoic acid methyl ester) by the recombinant *Escherichia coli* expressing the alkane hydroxylase (e.g., AlkBGT) and alkane transporter (AlkL) of *Pseudomonas putida* GPo1 [16,17]. 1,12-Dodecanedioic acid methyl ester was produced to a concentration of 117 mM by using a two-liquid phase whole-cell reaction system having bis(2-ethylhexyl) phthalate as organic carrier solvent [17].

**Scheme 1:**
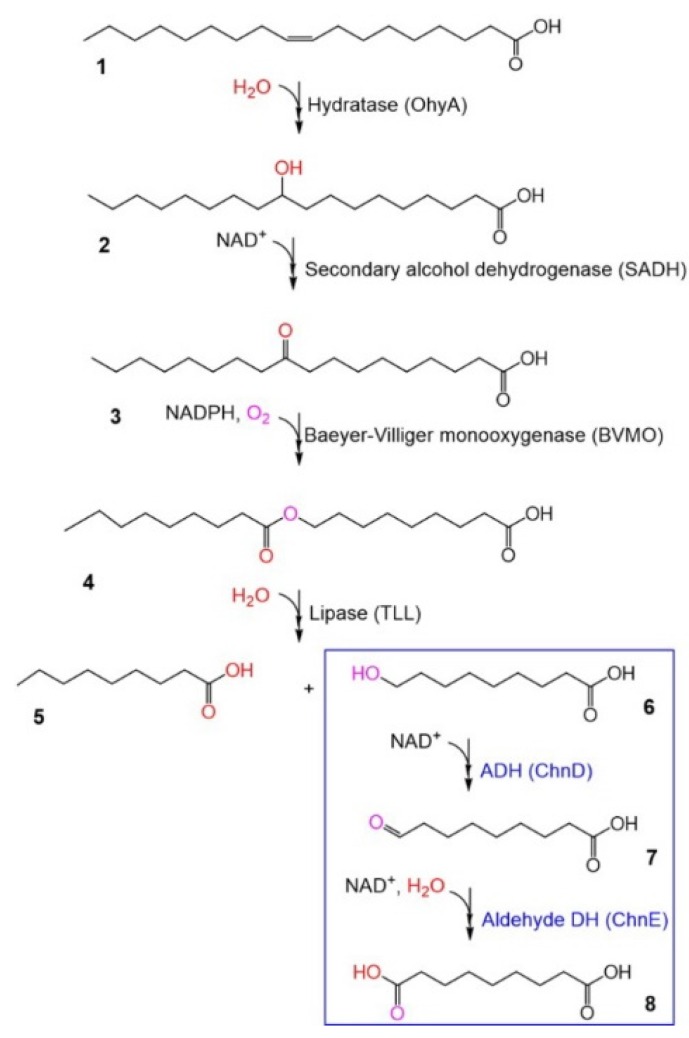
Designed biotransformation pathway. Oleic acid (**1**) is converted into *n*-nonanoic acid (**5**) and 1,9-nonanedioic acid (azelaic acid) (**8**) via 9-hydroxynonanoic acid (**6**) by cascade enzymes. The biotransformation pathway was constructed based on our previous studies [10,13,14].

Another example may include the production of ω-hydroxycarboxylic acids, α,ω-dicarboxylic acids and ω-aminocarboxylic acids via oxidative cleavage of a C–C bond of long chain fatty acids (e.g., oleic acid and ricinoleic acid) [10,13,14,15,18,19,20,21]. 9-Hydroxynonanoic acid could be produced from oleic acid or ricinoleic acid by the recombinant *E. coli* expressing a fatty acid double bond hydratase (OhyA) from *Stenotrophomonas maltophilia*, a secondary alcohol dehydrogenase from *Micrococcus luteus*, a Baeyer–Villiger monooxygenase (BVMO) from *Pseudomonas putida* KT2440, and a long chain fatty acid transporter (FadL) and a lipase from *Thermomyces lanuginosus* (Scheme 1) [10,18,19,20,22]. 9-Hydroxynonanoic acid was converted into 1,9-nonanedioic acid (i.e., azelaic acid) by the recombinant *E. coli* expressing alcohol/aldehyde dehydrogenases (ChnDE) from *Acinetobacter* sp. NCIMB 9871 [10]. Additionally, 9-hydroxynonanoic acid was transformed into 9-aminononanoic acid by a serial reaction of a primary alcohol dehydrogenase from *P. putida* GPo1 and ω-transaminase of *Silicibacter pomeroyi*. [14,15,23].

The enzymatic synthesis of polyesters and polyamides from biomass-derived monomers was extensively investigated with increasing attention as an eco-friendly approach for producing sustainable materials from renewable resources, and reducing greenhouse gas emissions and carbon footprint [24,25,26,27,28]. For instance, a series of bio-based alternatives based on 2,4-, 2,5-, and 2,6-pyridinedicarboxylic acid-derived polymers were produced via enzymatic catalysis [25]. In addition, the medium chain fatty acids (e.g., 9-hydroxynonanoic acid, 1,10-decanedioic acid, 1,11-undecanedioic acid) have been used for the production of esters and polymers [29,30,31,32,33].

In this study, *Corynebacterium glutamicum* was engineered to express the ChnDE of *Acinetobacter* sp. NCIMB 9871 [34] for the production of 1,9-nonanedioic acid from 9-hydroxynonanoic acid, which had been produced from oleic acid by the recombinant *E. coli* BL21(DE3) expressing the OhyA *of S. maltophilia*, the secondary alcohol dehydrogenase of *M. luteus*, the BVMO of *P. putida* KT2440, and the fadL (Scheme 1) [10,19]. Moreover, the enzymatic production of polyesters from 1,9-nonanedioic acid and 1,8-octanediol was investigated.

## 2. Materials and Methods

### 2.1. Bacterial Strains and Culture Conditions

*Escherichia coli* DH5α and *Corynebacterium glutamicum* ATCC 13032 were used for cloning and production of 1,9-nonanedioic acid, respectively. *E. coli* DH5α was grown in 5 mL Luria–Bertani (LB) medium (Becton, Dickinson and Company, Sparks, MD, USA) containing 10 g/L tryptone, 5 g/L yeast extract, and 10 g/L NaCl at 37 °C and 250 rpm. The recombinant *C. glutamicum* expressing the *Acinetobacter* sp. NCIMB 9871 alcohol/aldehyde dehydrogenase was cultivated in brain–heart infusion (BHI) medium (Becton, Dickinson and Company, Sparks, MD, USA) and supplemented with 25 µg/mL kanamycin at 30 °C for 12 h. The seed culture was inoculated (at 1% *v*/*v*) into 100 mL of CGXII medium containing 25 µg/mL kanamycin in a 500 mL baffled flask. The flask was cultivated at 30 °C for 12 h with shaking at 200 rpm. The CGXII medium contained 20 g/L (NH_4_)_2_SO_4_, 5 g/L urea, 1 g/L KH_2_PO_4_, 1 g/L K_2_HPO_4_, 0.25 g/L MgSO_4_·7H_2_O, 10 mg/L MnSO_4_·H_2_O, 1 mg/L ZnSO_4_·7H_2_O, 0.2 mg/L CuSO_4_, 0.02 mg/L NiCl_2_·6H_2_O, 0.2 mg/L biotin (pH 7.0), 40 g/L glucose, and 0.03 mg/L protocatechuic acid.

### 2.2. Azelaic Acid Tolerance Assay

*E. coli* BL21(DE3) and *C. glutamicum* ATCC 13032 wild type strains were used for the azelaic acid toxicity experiments. The assay was performed by adding azelaic acid at concentrations of 0–20 mM into the growth medium when the OD_600_ reached 0.5. *E. coli* was cultivated in LB and Riesenberg medium [35] at 37 °C with shaking at 250 rpm, and *C. glutamicum* was cultivated in BHI and CGXII medium at 30 °C with shaking at 200 rpm. Samples were taken periodically for the measurement of OD_600_.

### 2.3. Chemicals and Reagents

9-Hydroxynonanoic acid was purchased from Combi Blocks (Combi Blocks, San Diego, CA, USA) or prepared in our lab based on the previous study [10,13,19,20]. 1,9-Nonanedioic acid was obtained from Sigma-Aldrich (Sigma-Aldrich, St. Louis, MO, USA) or prepared in our lab based on the previous study [10,20]. 10-Hydroxydecanoic acid, 11-hydroxyundecanoic acid, 12-hydroxydodecanoic acid, 1,6-hexanediol, 1,10-decanedioic acid, 1,11-undecanedioic acid were obtained from Sigma-Aldrich (Sigma-Aldrich, St. Louis, MO, USA) and molecular sieves (3 Å, 4–8 mesh) were obtained from Sigma-Aldrich (Sigma-Aldrich, Steinheim, Germany). N-methyl-N-(trimethylsilyl) trifluoroacetamide (TMS) and 1,8-octanediol were supplied by TCI (TCI, Tokyo, Japan). Ethyl acetate (EA) and tetrahydrofuran (THF) were purchased from Duksan (Duksan, Ansan, South Korea). Tween 80 and toluene (99.5%) were obtained from Samchun (Samchun, Pyeongtaek, South Korea). GF CalB-IM™ (IM CALB) (specific activity: 1200 BOU/g (BOU *=* Butyl Oleate Unit)), consisting of Candida antarctica lipase B (CALB) that is bound to the microporous ion exchange resin by adsorption, was provided by GenoFocus (GenoFocus, Daejeon, South Korea).

### 2.4. Plasmid Construction

The *E. coli*/*C. glutamicum* shuttle vector, pCES208H36GFP, a derivative of pCES208 (KAIST, Daejeon, Korea) was used for cloning. The *chnE* and *chnD* genes of *Acinetobacter* sp. NCIMB 9871 [34] were inserted to pCES208H36GFP vector, resulting in a recombinant pCES208H36GFP-ChnDE. The *chnE* was amplified by a polymerase chain reaction (PCR) and inserted into the BamHI and NdeI restriction sites of pCES208H36GFP vector using the Infusion cloning kit (Takara Bio, Mountain View, CA, USA). The *chnD* was ligated into the HpaI site of the vector containing the *chnE*. The forward and reverse primers used for *chnE* and *chnD* amplification are shown in Table S1. All PCR constructs were confirmed by sequencing (Cosmogenetech Co., Seoul, Korea). The constructed vector was transformed into *C. glutamicum* by electroporation using a MicroPulser electroporator (BioRad, Hercules, CA, USA).

### 2.5. Whole-Cell Biotransformation

The biotransformations were conducted based on our previous reports [9,10,36,37,38]. Briefly, the recombinant cells were harvested at the stationary growth phase usually 12 h after cell cultivation by centrifugation at 4 °C. The cells were washed and resuspended into 50 mM Tris–HCl (pH 8.0) buffer. The whole-cell bioconversion was initiated by adding 9-hydroxynonanoic acid (**6**) in 50 mM Tris–HCl buffer (pH 8.0) containing 8 g dry cells/L and 0.5 g/L Tween 80 at 35 °C with agitation speed of 200 rpm.

### 2.6. Polymerization of Azelaic Acid for the Synthesis of Bio-Based Polyesters

Polymerization for the synthesis of bio-based polyesters were conducted by using 1,9-nonanedioic acid (3.76 g, 20 mmol), 1,6-hexanediol (2.36 g, 20 mmol), and 1,8-octanediol (2.925 g, 20 mmol). The polymerization reactions were performed in a 2-neck round bottom flask (250 mL flask) containing toluene (3:1 *v*/*w* of total monomers) as a solvent with a magnetic stirrer. IM-CALB (3% *w*/*w* of total monomers) and molecular sieves (6.25 g) were added into a flask which was capped with a rubber septum. The reaction flask was placed into a stirring mantle at 75 °C and 1000 rpm under constant pressure. The samples were obtained at 24, 48, and 72 h. The reactions were terminated by filtering enzymes and molecular sieves with membrane filter. Then, the resulting products were isolated to a white powder via vacuum evaporation. 

When diphenyl ether (2:1 *v*/*w* of total monomers) was used as a solvent, the polymerization reactions were performed with three kinds of diacid monomers which were 1,9-nonanedioic acid (0.47 g, 2.5 mmol), 1,10-decanedioic acid (0.5 g, 2.5 mmol), and 1,11-undecanedioic acid (0.55 g, 2.5 mmol) and polyol (e.g., 1,8-octanediol (0.36 g, 2.5 mmol)). Molecular sieves (0.25 g), GF CalB-IM™ (3% *w*/*w* of total monomers), and the monomers (2.5 mmol diacid/diol) were added into a 4 mL glass vial and reacted in a thermomixer at 75 °C and 500 rpm. The samples were taken at 24, 48, and 72 h, diluted with THF and filtered with a PTFE syringe filter (0.45 μm). Sample solutions were analyzed by gel permeation chromatography (GPC) to determine the number-average molecular weight (*Mn*).

### 2.7. GC/MS Analysis

The concentrations of the remaining fatty acids and accumulating carboxylic acids in the medium (e.g., 9-hydroxynonanoic acid (6), 1,9-nonanedioic acid (8)) were determined as described previously [13,19,20]). The reaction medium was mixed with an equal volume of ethyl acetate containing 0.5 g/L methyl palmitate as an internal standard. The organic phase was harvested after vigorous vortexing and subjected to derivatization with N-methyl-N-(trimethylsilyl) trifluoroacetamide. The trimethylsilyl derivatives were analyzed using a Thermo Ultra Trace GC system connected to an ion trap mass detector (Thermo ITQ1100 GC-ion Trap MS, Thermo Scientific, Indianapolis, IN, USA). The derivatives were separated on a nonpolar capillary column (30 m length, 0.25 mm film thickness, HP-5MS, Agilent Technologies, Palo Alto, CA, USA). A linear temperature gradient was programmed as 160 °C, 25 °C/min to 235 °C, and 3 °C/min to 253 °C. The injection port temperature was 230 °C. Mass spectra were obtained within the range of 100–600 m/z. Selected ion monitoring was used for detection and the fragmentation analysis of the reaction products.

### 2.8. Gel Permeation Chromatography Analysis

The number-average molecular weight (*Mn*) of samples was analyzed by gel permeation chromatography (GPC) using a Waters HPLC system equipped with a Waters 1525 Binary HPLC Pump, Waters 2414 Refractive Index Detector, and Waters 2707 Autosampler. The Breeze2 HPLC system was used for the software program. THF was used as an eluent at a flow rate of 1.0 mL/min at 35 °C. Injection volumes of 50 μL (0.2% of a concentration) were used and the total running time was 30 min. HR3 Styragel and HR4 Styragel coluMns were used for GPC. The molecular weight range of the former was 500 to 30,000 and the latter was 5,000 to 60,0000. The size of the particle was 5 μm and the thickness and length of the column was 7.8 mm and 300 mm.

### 2.9. Characterization of Polyesters Using Fourier-Transform Infrared Spectroscopy and ^1^H-NMR Analysis

The polymerization of 1,9-nonanedioic acid and 1,8-octanediol was monitored by assessing the transformation of functional groups such as COOH, OH, C=O, and C–O–C by Fourier-Transform Infrared Spectroscopy (FT-IR). The spectra of samples were obtained in a region of 500–4000 cm^−1^ using Nicolet-iS5 (Thermo Scientific, Medison, WI, USA). An air background spectrum was collected before the analysis of a sample and subtracted from each sample spectrum. The structure of the product was elucidated in CDCl_3_ by ^1^H-NMR using 400 MHz NMR (Bruker, UltraShield Plus 400, Billerica, MA, USA). 

## 3. Results and Discussion

### 3.1. Construction of the Recombinant C. Glutamicum-Based Biocatalyst

**Figure 1:**
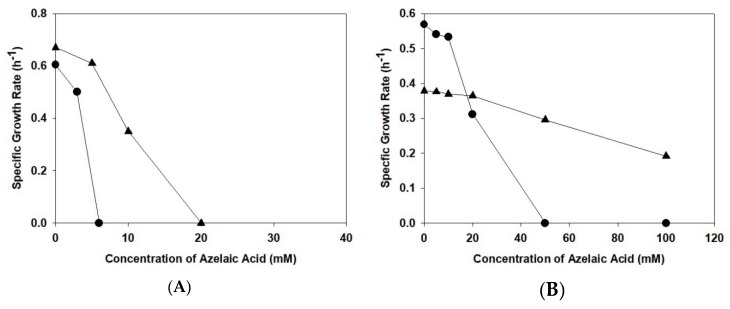
Effect of azelaic acid concentration on the specific growth rates of *Escherichia coli* BL21 and *Corynebacterium glutamicum* in a complex medium (Luria–Bertani (LB) or brain–heart infusion (BHI) medium) (**A**) and a glucose mineral medium (Riesenberg or CGXII medium) (**B**). After entering exponential growth, a 100-mL culture was split into 20-mL subcultures, to which a different amount of azelaic acid was added for the toxicity experiments. Symbols indicate the specific growth rates of *E. coli* BL21 (●) and *C. glutamicum* (▲).

1,9-Nonanedioic acid (i.e., azelaic acid) has been reported to be very toxic to microbial cells. Thereby, its toxicity to a representative Gram (-) bacteria (i.e., *E. coli* BL21) and Gram (+) bacteria (i.e., *C. glutamicum* ATCC 13032) was first investigated. The cell growth of *E. coli* BL21 in the LB medium was completely inhibited in the presence of 6 mM azelaic acid (Figure 1A). On the other hand, *C. glutamicum* ATCC 13032 was able to grow in the BHI medium containing almost 20 mM azelaic acid (Figure 1A). Notably, *C. glutamicum* displayed stronger tolerance against toxic effects of azelaic acid in the glucose mineral medium (i.e., CGXII medium) (Figure 1B). Thus, *C. glutamicum* ATCC 13032 was chosen as the host cell for the production of azelaic acid.

The alcohol/aldehyde dehydrogenases (ChnDE) of *Acinetobacter* sp. NCIMB 9871 were reported to have a great activity with respect to oxidation of 9-hydroxynonanoic acid (6) into azelaic acid (8) [10]. Thereby, the *chnDE* had been inserted into an *E. coli*/*C. glutamicum* shuttle vector (i.e., pCES208H36GFP [39,40]) (Figure S1). Here, one of the key points for construction of the gene expression vector was to make the expression level of ChnE greater than that of ChnD, because the ChnD reaction product (7) is very toxic to microbial cells. When the resulting plasmid (i.e., pCES208H36GFP-ChnDE) was introduced into *C. glutamicum* ATCC 13032, the recombinant enzymes ChnDE were expressed in *C. glutamicum* pCES208H36GFP-ChnDE to a rather high degree in a soluble form (Figure S2). The expression level of ChnE was markedly higher than that of ChnD.

### 3.2. Biosynthesis of 1,9-Nonanedioic Acid from 9-Hydroxynonanoic Acid 

The biotransformation activity of *C. glutamicum* pCES208H36GFP-ChnDE was examined by conducting whole-cell biotransformation of 9-hydroxynonanoic acid (6) into azelaic acid (8). 9-Hydroxynonanoic acid (6), which had been produced from oleic acid (1) (purity: ca. 80%) by a lipase and a recombinant *E. coli* BL21(DE3) expressing a fatty acid double-bond hydratase (OhyA) from *Stenotrophomonas maltophilia*, a long-chain secondary alcohol dehydrogenase from *Micrococcus luteus*, and an engineered Baeyer–Villiger monooxygenase (BVMO) from *Pseudomonas putida* KT2440 (Scheme 1) [10,13,19], was used as the reaction substrate.

**Figure 2:**
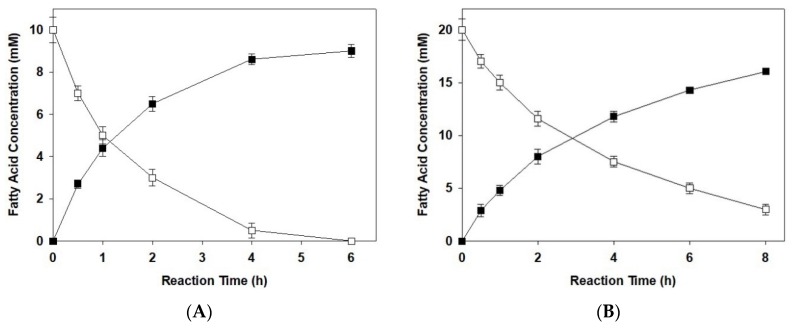
Time course of the biotransformation of 9-hydroxynonanoic acid (**6**) into azelaic acid (**8**). The biotransformation was initiated by adding 10 mM (**A**) or 20 mM 9-hydroxynonanoic acid (**B**) into 50 mM Tris–HCl buffer (pH 8.0) containing 8 g dry cells/L of *C. glutamicum* pCES208H36GFP-ChnDE and 0.5 g/L Tween 80 at 35 °C. Symbols indicate concentrations of 9-hydroxynonanoic acid (**6**) (□) and azelaic acid (**8**) (■). All experiments were performed in triplicate. The error bars indicate standard deviations.

After cultivation of the recombinant *C. glutamicum* pCES208H36GFP-ChnDE in the CGXII medium to the stationary growth phase, they were recovered by centrifugation and resuspended into 50 mM Tris–HCl buffer (pH 8.0). When 9-hydroxynonanoic acid was added to a concentration of 10 mM in the reaction medium containing 8 g dry cells/L and 0.5 g/L Tween 80, azelaic acid accumulated to 9 mM at t = 4 h (Figure 2A). The toxic reaction intermediate was not observed in the reaction medium, indicating that the catalytic activity of ChnE was greater than that of ChnD. The specific biotransformation rate and conversion yield were increased up to 12 U/g dry cells and 90%, respectively. The oxidation products (**8**) were not detected when the C. glutamicum, which had been transformed with an empty vector, was used. Furthermore, the addition of 20 mM 9-hydroxynonanoic acid into the reaction medium led to the production of azelaic acid to 16 mM at t = 8 h (Figure 2B). These results indicated that the recombinant biocatalyst is quite active and efficient with respect to oxidation of 9-hydroxynonanoic acid (**6**) into azelaic acid (**8**).

The recombinant *C. glutamicum* pCES208H36GFP-ChnDE was used for the production of other α,ω-dicarboxylic acids such as 1,10-decanedioic acid, 1,11-undecanedioic acid, and 1,12-dodecanedioic acid from corresponding ω-hydroxycarboxylic acids (Figure S3). When the whole-cell biotransformations were carried out under the conditions comparable to the experiment shown in Figure 2B, oxidation of 12-hydroxydodecanoic acid into 1,12-dodecanedioic acid exhibited the highest reaction rate. This indicated that the *C. glutamicum* pCES208H36GFP-ChnDE-biocatalyst could be used for the production of a variety of α,ω-dicarboxylic acids.

### 3.3. Production of Polyesters from α,ω-Diacids and α,ω-Diols

The resulting azelaic acid isolated from the reaction medium was used for polycondensation with α,ω-diols such as 1,6-hexanediol and 1,8-octanediol by using the immobilized lipase B from *Candida antarctica* (i.e., CalB-IM), which have been the most commonly employed biocatalysts for various (trans)esterifications and polyesterifications [24,25,26,27,41]. The experiments were designed on a basis of the polymerizations of adipic acid with 1,4-butanediol, 1,6-hexanediol, and 1,8-octanediol [42], esterification of polyol with dimethylcarbonate [43,44], and ring opening polymerization of itaconic acid by Novozyme 435 [45]. An important factor for obtaining high biocatalyst performance, the effects of solvents were investigated using toluene and diphenyl ether on the molecular weight of product in the reaction time course. Although water is one of the desirable solvents, organic solvents are often required to dissolve reactants for homogeneous reaction conditions. Toluene (log P = 2.68) is classified as a usable solvent concerning the green chemistry principle of efficiency in reaction design [46]. Generally, the higher enzyme activity shows in hydrophobic solvents (with a high log P value) than in hydrophilic solvents (with a low log P value), while the hydrophilic solvents are used to dissolve more polar solid substrate [45,47]. The CalB-IM-catalyzed polymerization was carried out in the solvents at 75 °C with 3 wt % catalyst to the monomers in the presence of molecular sieves as scavengers of resulting H_2_O. 

**Figure 3:**
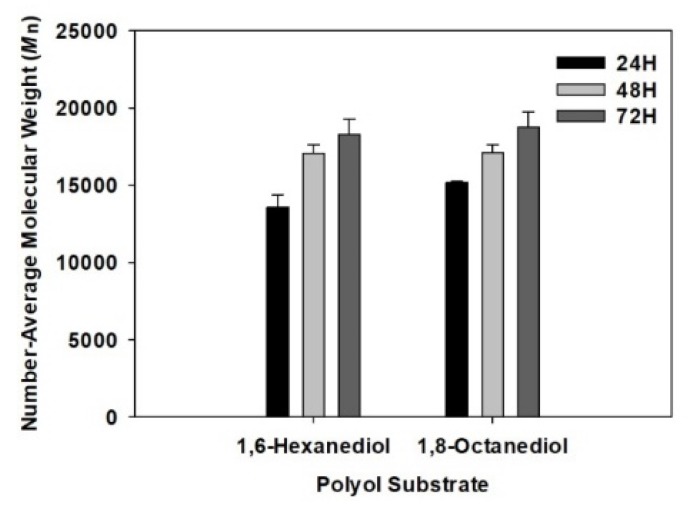
Polymerization reaction of azelaic acid and 1,6-hexanediol or 1,8-octanediol by the immobilized lipase B from *Candida antarctica* (i.e., GF CalB-IM (GenoFocus (Korea))). The reaction was initiated by adding CalB-IM to a concentration of 3% (*w/w*) catalyst/monomer in toluene (3:1 *v/w* of total monomers) containing a molecular sieve and the reaction substrates. The reaction temperature and agitation were maintained at 75 °C and 1000 rpm. All experiments were performed in triplicate. The error bars represent standard deviation.

**Figure 4:**
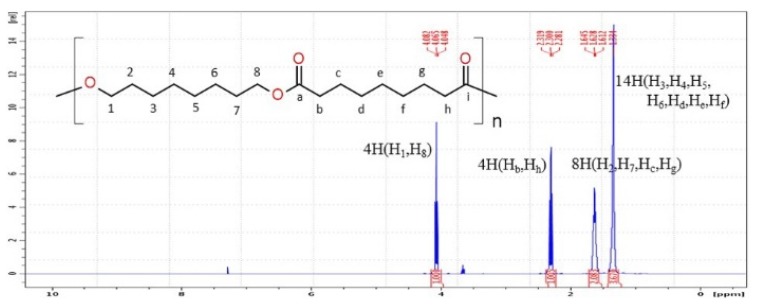
^1^H-NMR (400 MHz, CDCl_3_) spectrum of polyester prepared from the reaction of 1,8-octanediol and azelaic acid in toluene at 75 °C.

The progress of the reactions was examined by GPC analysis of aliquots withdrawn from the reactions after 24, 48, and 72 h. The results showed the increased molecular weight over reaction times (Figure 3). The number-average molecular weight (*M*_n_) of the products from azelaic acid with 1,6-hexanediol and 1,8-octanediol increased up to 20,000 at t = 72 h, respectively. The polyester products were not detected when the reaction was conducted without IM-CALB. The resulting products were isolated to a white powder via in vacuo evaporation (Figure S4). FTIR spectra confirmed the peak changes of the functional groups in the synthesis of the polyesters from azelaic acid and 1,8-octanediol (Figure S5A). After polyesterification, a new strong peak of the stretching vibration bands of C=O at 1726 cm^−1^ (Figure S5C) was observed as the formation of ester bond, while the acid peaks of C=O (1689 cm^−1^) and OH (broad 2500–3500 cm^−1^) in Figure S5B and the diol peaks of OH (3391 and 3324 cm^−1^) in Figure S5A were completely disappeared in the resulting polymer product (Figure S5C). Additionally, the formation of the linear aliphatic C–O–C group from acid and alcohol provided a strong new peak at 1170 cm^−1^ (Figure S5C). This reaction was quantitatively carried out and completed to form polyesters in the condition. Furthermore, the structure of polyester prepared was clearly elucidated by ^1^H-NMR analysis (Figure 4). The ester bond (–CO–OCH_2_–) formed by polymerization of 1,8-octanediol and azelaic acid appeared as a triplet peak at δ4.065 (4H).

**Figure 5:**
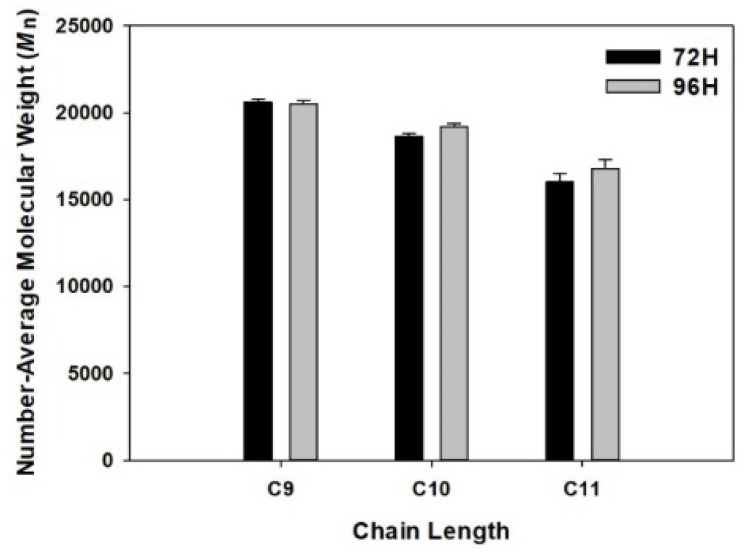
Polymerization reaction of azelaic acid, sebacic acid, and 1,11-undecanedioic acid with 1,8-octanediol by CalB-IM. The reaction was initiated by adding CalB-IM to a concentration of 3% (*w/w*) catalyst/monomer in biphenyl ether (2:1 *v/w* of total monomers) containing a molecular sieve and the reaction substrates. The reaction temperature and agitation were maintained at 75 °C and 1000 rpm. All experiments were performed in triplicate. The error bars represent standard deviation.

With an aim to investigate the effects of solvents, the CalB-IM-catalyzed condensation reaction of azelaic acid and 1,8-octanediol was conducted in diphenyl ether, which allowed the highest *M*_n_ of the product from the polyesterification of adipic acid and 1,8-octanediol among four solvents (diphenyl ether, xylene, tetraethylene glycol dimethyl ether, and 2-methoxyethyl ether) in the previous study [48]. The polymerization of azelaic acid and 1,8-octanediol in diphenyl ether led to slightly greater *M*_n_ and *M*_w_ values (i.e., 21,000 and 27,000 at t = 72 h) as compared to the polyesterification in toluene shown in Figure 3 (Figure 5 and Table 1). These results indicated that the molecular characteristics of the polymers were influenced by the solvents used. We will consider using alternative greener solvents such as 2,2,5,5-tetramethyloxolane, 2-methyltetrahydrofuran, 2,5-dimethyltetrahydrofuran, 2,2,5,5-tetramethyltetrahydrofuran, and pinacolone, which have been recently reported [49,50], for further studies.

Additionally, CalB-IM-catalyzed polymerization of longer chain length α,ω-dicarboxylic acids with 1,8-octanediol in diphenyl ether was conducted in order to examine the effects of chain length of α,ω-dicarboxylic acids on the molecular weight of the products (Figure 5). The longer chain of α,ω-dicarboxylic acids, 1,10-decanedioic acid, and 1,11-undecanedioic acid resulted in the lower Mn and M_w_ values and lower polydispersity of product than shorter chain azelaic acid (Table 1). This result suggested that the CalB-IM-catalyzed polyesterification of α,ω-dicarboxylic acids and 1,8-octanediol was influenced on the molecular weight by the chain length of α,ω-dicarboxylic acids used.

## 4. Conclusions

This study has demonstrated that the toxic azelaic acid could be produced from a renewable fatty acid (oleic acid) by using the recombinant *C. glutamicum*, expressing the alcohol/aldehyde dehydrogenases (ChnDE) of *Acinetobacter* sp. NCIMB 9871. Moreover, the dicarboxylic acid could be used for the production of long chain biopolyester with the *M*_n_ of approximately 21,000 by an immobilized lipase. We will conduct further studies to increase molecular weight of the long chain biopolyesters.

## Figures and Tables

**Table 1:** Molecular weight and polydispersity of the polyesters produced by CalB-IM.

	Solvent	Diacid	Diol	*M_n_* (Da)	*M*_w_ (Da)	Polydispersity
**1**	Toluene	Azelaic acid	1,6-Hexanediol	20,000	25,000	1.26
**2**			1,8-Octanediol	20,000	25,000	1.27
**3**	Diphenyl ether	Azelaic acid	1,8-Octanediol	21,000	27,000	1.26
**4**		Sebacic acid	1,8-Octanediol	19,000	24,000	1.25
**5**		1,11-Undecanedioic acid	1,8-Octanediol	17,000	21,000	1.21

## Supplementary Materials

The following are available online at https://www.mdpi.com/2073-4360/11/10/1690/s1, Figure S1: Map of pCES208H36GFP-ChnDE for the ChnDE expression in *Corynebacterium glutamicum* ATCC 13032, Figure S2: SDS-PAGE analysis of the protein extracts of *C. glutamicum* ATCC 13032 and the recombinant *C. glutamicum* ATCC 13032 pCES208H36GFP-ChnDE, Figure S3: The specific oxidation rates of the recombinant *C. glutamicum* for the C9 to C12 ω-hydroxycarboxylic acids, Figure S4: HPLC chromatogram of the biopolyester, which had been produced from azelaic acid and 1,8-octanediol by the immobilized lipase B from *Candida antarctica* (i.e., GF CalB-IM (GenoFocus (Korea))) (A). The biopolyester, which had been isolated from the reaction medium (B), Figure S5: FT-IR spectra of the reaction components, (A) 1,8-octanediol and (B) azelaic acid, and (C) polyester product formed in the poly-esterification in toluene at 75 °C, Table S1: Bacterial strains, plasmids, and oligonucleotides used in this study.
