# Supplementary material for: Multi-Step Enzymatic Synthesis of 1,9-Nonanedioic Acid from a Renewable Fatty Acid and Its Application for the Enzymatic Production of Biopolyesters
Source: Polymers (Basel). 2019 Oct 15;11(10):1690. doi: 10.3390/polym11101690 (PMC6835665; doi:10.3390/polym11101690)
Supplement: Supplementary file 1 [file polymers-11-01690-s001.docx]

Supplementary File

Multi-step Enzymatic Synthesis of 1,9-Nonanedioic Acid from a Renewable Fatty Acid and Its Application for the Enzymatic Production of Biopolyesters

Hyun-Ju Lee^1,†^, Young-Seo Kang^1, †^, Chae-Yun Kim^1^, Eun-Ji Seo^1^, Sang-Hyun Pyo^2^, and Jin-Byung Park^1,*^

^1^ Department of Food Science & Engineering, Ewha Womans University, Seoul 03760, Republic of Korea

^2^ Biotechnology, Department of Chemistry, Center for Chemistry and Chemical Engineering, Lund University, SE-22100 Lund, Sweden

***** Correspondence: [jbpark06@ewha.ac.kr](mailto:jbpark06@ewha.ac.kr); Tel.: +82-2-3277-4509

† Hyun-Ju Lee and Young-Seo Kang contributed equally to this work.

**Figure S1.** Map of pCES208H36GFP-ChnDE for the ChnDE expression in *Corynebacterium glutamicum* ATCC 13032. The alcohol/aldehyde dehydrogenase genes (chnDE) of *Acinetobacter* sp. NCIMB 9871 [1] were inserted into an *E. coli*/*C. glutamicum* shuttle vector, pCES208H36GFP [2, 3].


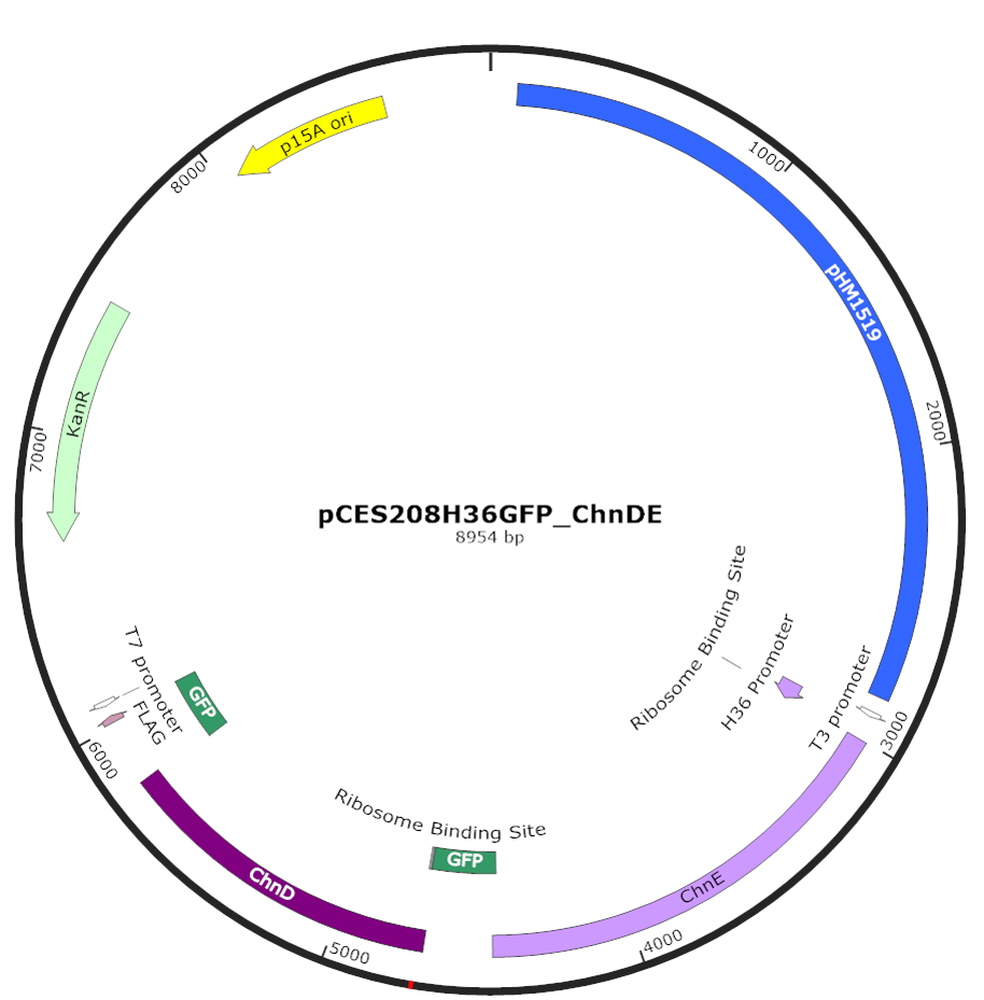


**Figure S2.** SDS-PAGE analysis of the protein extracts of *C. glutamicum* ATCC 13032 and the recombinant *C. glutamicum* ATCC 13032 pCES208H36GFP-ChnDE. The wild type (lanes 1,2,3) and recombinant cells (lanes 4,5,6) were harvested after 12 h of cultivation in CGXII medium and fractionated to total, soluble and insoluble fractions. Lane M: marker protein; lanes 1,4: total fraction; lanes 2,5: soluble fraction; lanes 3,6: insoluble fraction.

**
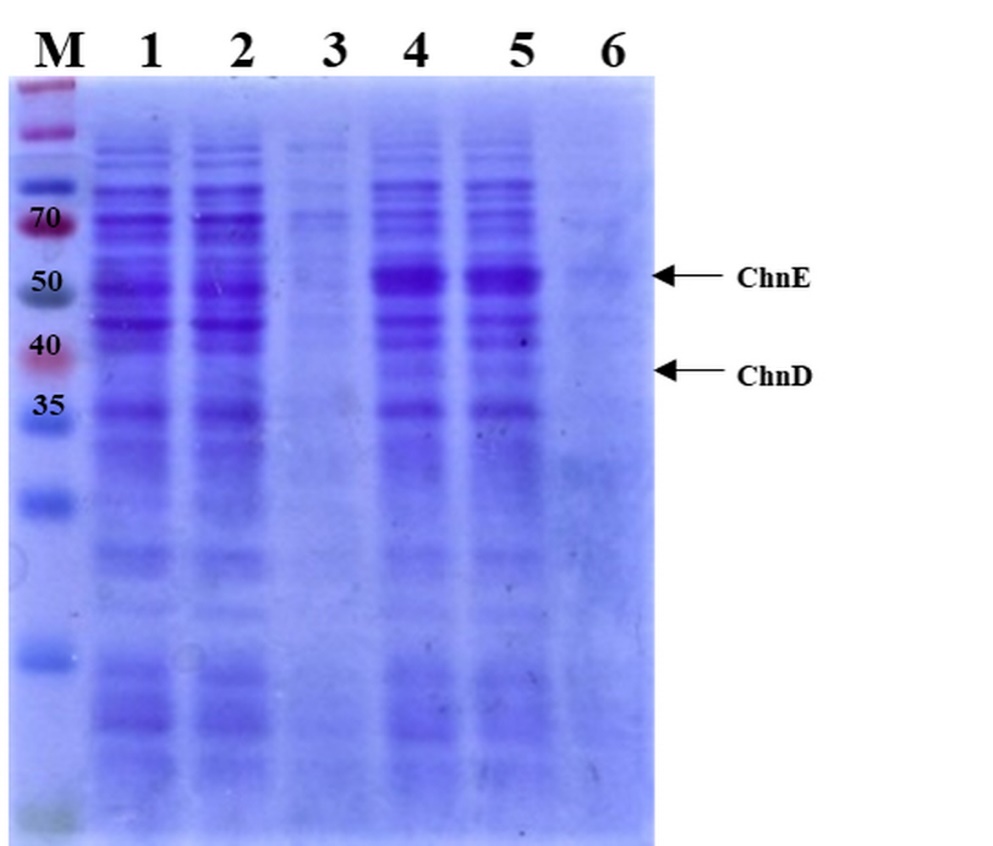
**

**Figure S3.** The specific oxidation rates of the recombinant *C. glutamicum* for the C9 to C12 ω-hydroxycarboxylic acids. The whole-cell bioconversion was initiated by adding 20 mM substrate (e.g., 9-hydroxynonanoic acid, 10-hydroxydecanoic acid, 11-hydroxyundecanoic acid, 12-hydroxydodecanoic acid), which were prepared in dimethyl sulfoxide (DMSO). The reaction was conducted in 50 mM Tris-HCl buffer (pH 8.0) containing 8 g dry cells/L and 0.5 g/L Tween 80 at 35°C, 200 rpm. The specific oxidation rates were determined based on the product concentrations at 10 or 30 min.


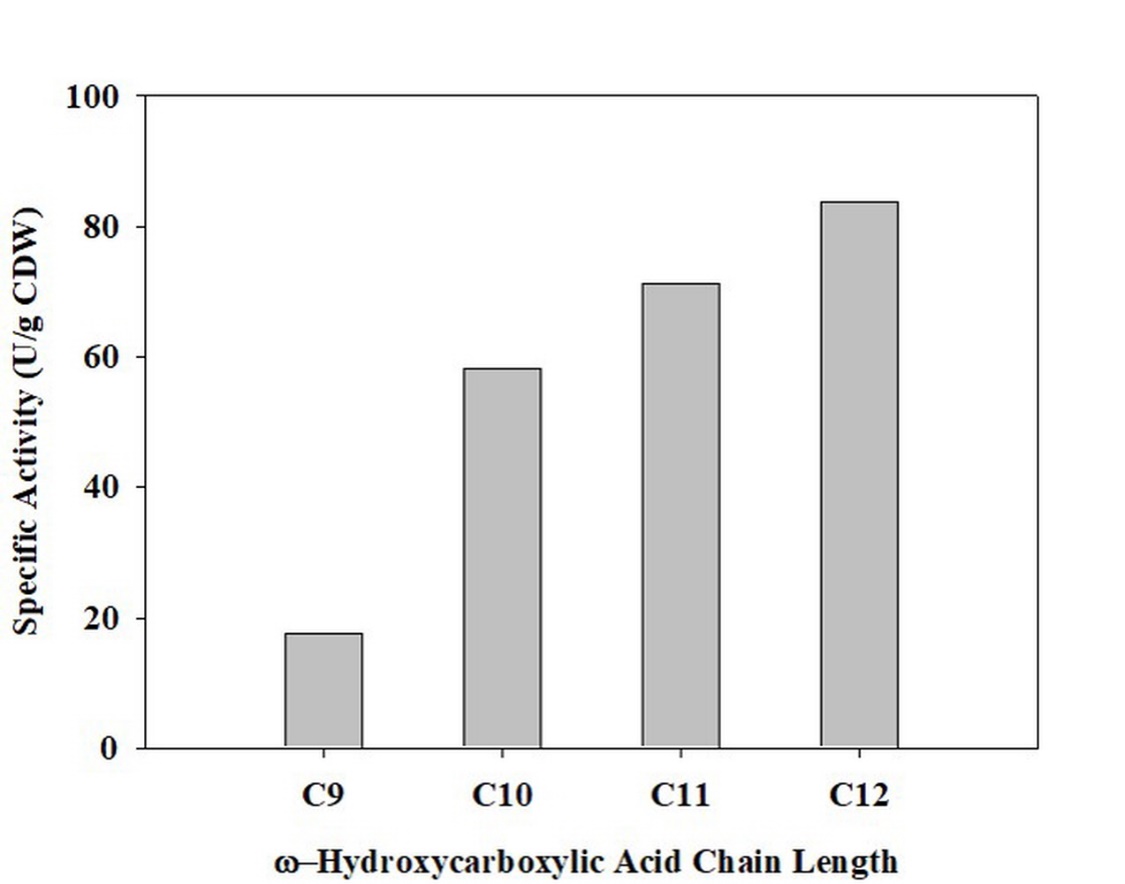


**Figure S4.** HPLC chromatogram of the biopolyester, which had been produced from azelaic acid and 1,8-octanediol by the immobilized lipase B from *Candida antarctica* (i.e., GF CalB-IM (GenoFocus (Korea))) (**A**). The biopolyester, which had been isolated from the reaction medium (**B**).

(**A**)


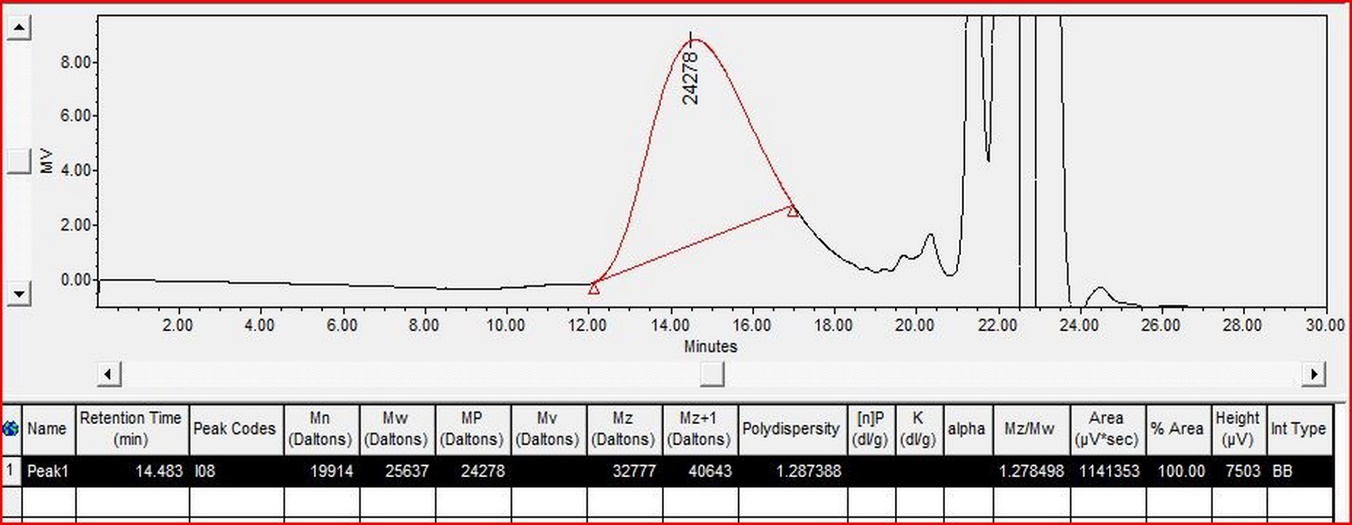


(**B**)


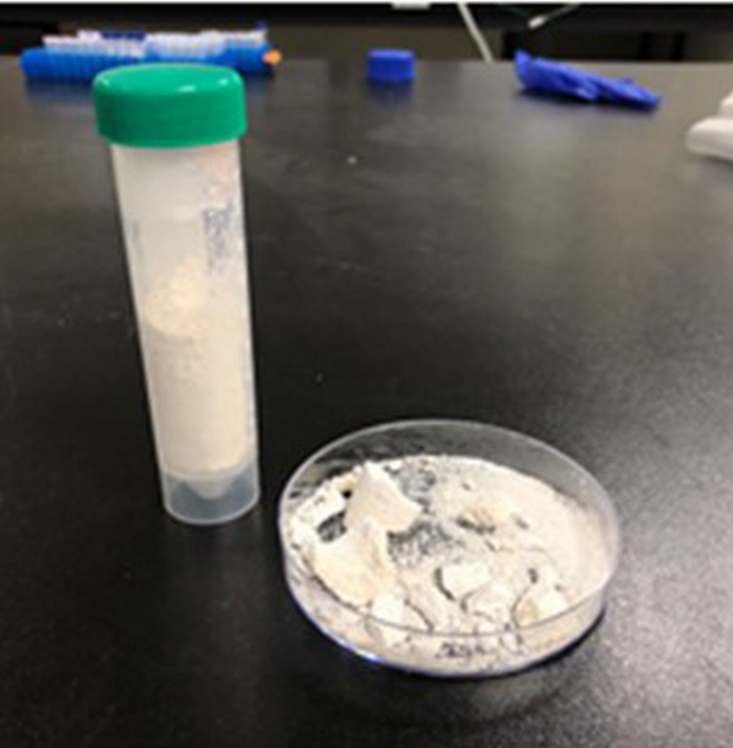


**Figure S5.** FT-IR spectra of the reaction components, (A) 1,8-octanediol and (B) azelaic acid and (C) polyester product formed in the poly-esterification in toluene at 75°C.


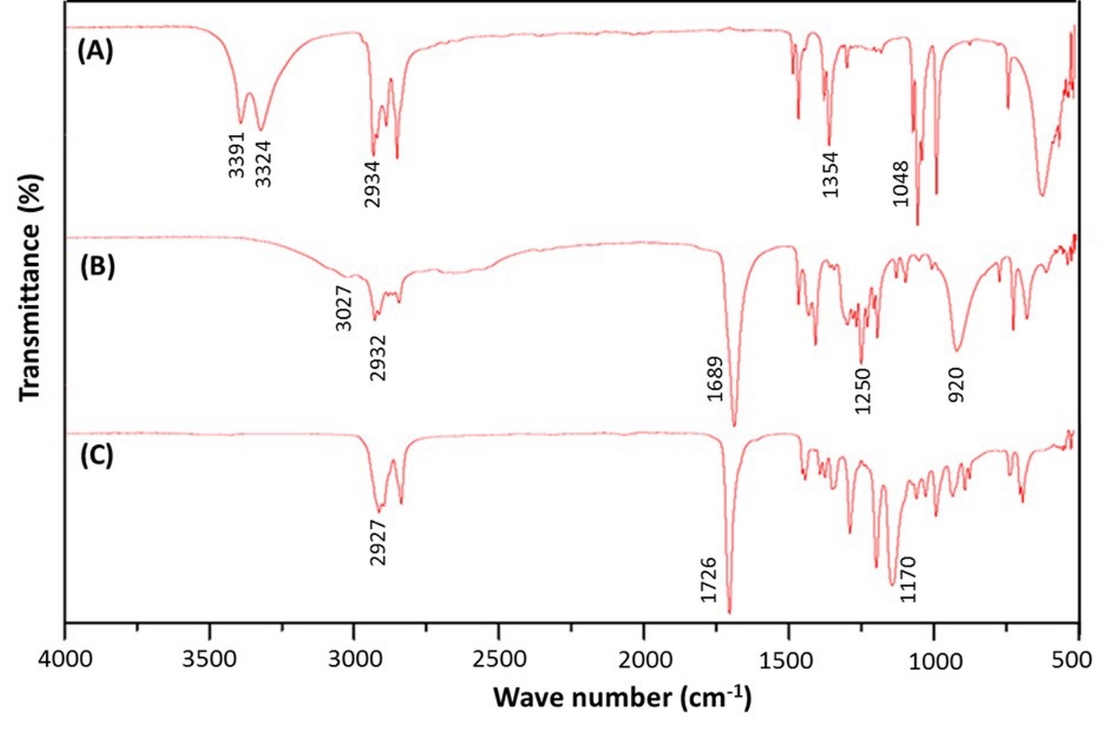


**Table S1.** Bacterial strains, plasmids, and oligonucleotides used in this study.

|  | Relevant characteristics | Reference or source |
| --- | --- | --- |
| **Strains**  *E. coli* DH5α  *C. glutamicum*  **Plasmids**  pCES208H36GFP  pCES208H36GFP-ChnDE  **Primers**  *ChnE*-F  *ChnE*-R  *ChnD*-F  *ChnD*-R | F- (80d lacZ M15) (lacZYA-argF) U169 hsdR17  (r - m +) recA1 endA1 relA1 deoR  Wild type  6.7 kb, *E. coli*-*C. glutamicum* shuttle vector, Km^r^, pCES208 derivative; P_H36_, eGFP  8.9 kb, pCES208 derivative; P_H36_, eGFP  5’-GAGTAGCATG**GGATCC**ATGAACTATCCA AATATACCTTTATATATCAACGGTGAG-3’  5’-TCATGCTGTTT**CATATG**CTAATTGAGTTG CGTAATAAATTTGGTTCTGAGGT-3’  5’- AATGGAATCAAA**GTT**AGAAAGGAGGAT GCACTGTTACTGCGTGACG-3’  5’- TCTAATTTTGAA**GTT**TCAGTTTTCGTGCA TAAGCACAATACG-3’ | RBC (Real Biotech)  ATCC 13032  [3]  This study  *Bam*HI  *Nde*I  *Hpa*I  *Hpa*I |

Restriction sites are shown in bold. The underlined nucleotides represent ribosome binding site.

References

1. Iwaki, H.; Hasegawa, Y.; Teraoka, M.; Tokuyama, T.; Bergeron, H.; Lau, P.C. Identification of a Transcriptional Activator (ChnR) and a 6-Oxohexanoate Dehydrogenase (ChnE) in the Cyclohexanol Catabolic Pathway in *Acinetobacter* sp. Strain NCIMB 9871 and Localization of the Genes that Encode Them. *J. Appl. Environ. Microbiol.* **1999**, *65*, 5158-5162.
2. Park, J.-U.; Jo, J.-H.; Kim, Y.-J.; Chung, S.-S.; Lee, J.-H.; Lee, H.-H. Construction of Heat-Inducible Expression Vector of *Corynebacterium glutamicum* and *C. ammoniagenes*: Fusion of λ Operator with Promoters Isolated from *C. ammoniagenes*. *J. Microbiol. Biotechnol.* **2008**, *18*, 639-647.
3. Yim, S.S.; An, S.J.; Kang, M.; Lee, J.; Jeong, K.J. Isolation of Fully Synthetic Promoters for High-Level Gene Expression in *Corynebacterium glutamicum*. *Biotechnol. Bioeng.* **2013**, *110*, 2959-2969, doi:10.1002/bit.24954.
